# Review of *What Are Zoos For?*

**DOI:** 10.1017/S0962728625100092

**Published:** 2025-06-20

**Authors:** Lisa Riley

**Affiliations:** Senior Lecturer in Animal Behaviour and Welfare, University of Winchester, UK

It is perhaps not surprising, given their industry and academic backgrounds, that Browning and Veit have chosen to write an animal welfare/animal rights-focused account of the role of modern zoos. *What Are Zoos For?* is a concise yet detailed account of what the very best zoos currently evidence and what all zoos should aspire to be – centres which safeguard animals for the purposes of conservation. Early in the Introduction, the authors neatly answer the title question; zoos, they declare, are *for animals.* By presenting the deontological argument of whether zoos are ethically justifiable, both advocacy and abolitionist reasoning are outlined to firmly establish this book in the advocacy camp. The authors set out their intentions to praise good zoos and highlight good practice (which some may argue should simply be *standard practice*) by examining the ‘four pillars’ or roles of modern zoos (rather than roadside zoos, menageries, or alike) and therefore – chapter by chapter – recreation, research, conservation, and education are discussed.

Recreation is, the authors state, a major ethical issue for zoos given the pivotal abolitionist argument that keeping wild animals in zoos for the purpose of entertainment is not justifiable. While the history of zoos as entertainment centres is summarised, as has been done in many texts before, the Recreation chapter offers a more detailed ethical evaluation of the possible harms and benefits of having human visitors at the zoo. Points on human superiority and domination over non-human animals could be explored further; most people still primarily go to the zoo for a good day out and while the zoo and its staff may not see animals as ‘less than humans’ much of wider society does. Interesting discussions on animal dignity are presented, with convincing evidence that zoos attempt to promote animal agency while balancing the wants and desires of human visitors to connect with nature.

Enter discussions on Research. Here, the authors are sympathetic to the needs of zoos (as businesses/organisations), to zoo animals, to visitors and to zoo academics, noting the need for research to guide better husbandry (what, in an abolitionist argument, may be considered a self-fulfilling prophecy), but also sharing myriad ways zoos engage with research to improve foundational biological knowledge and insight into animal cognition. Zoos can also be the site of human research, particularly on human knowledge, understanding and empathy for animals and the natural world. This research trajectory could be explored further though understandably the authors want to keep focus on zoo animals and their welfare.

Conservation is subsequently examined and its importance noted; it is often postulated that evidencing the conservation pillar of the modern zoo is the major reason why zoos should be considered ethically justifiable. With a now familiar structure, this chapter examines the conservation history of zoos with considered arguments for the role of zoos in *ex* and *in situ* conservation and with the authors challenging the notion that zoos take away conservation funds from direct conservation initiatives. The seasoned debate of individual welfare vs species conservation is explored with the authors unsurprisingly favouring welfare of the individual. While many impactful examples of zoo conservation action are discussed, one pivotal question remains unexplored – why so many species kept in zoos are of ‘least concern’ on the IUCN Red List matrix of conservation threat.

Maybe the education role of zoos justifies their existence? For people to care about the natural world (and hopefully take action to secure its future), they must first know about it! Zoos, the authors suggest, have many avenues of possibility to educate visitors and beyond. While Browning and Veit recognise the importance of zoo educational research demonstrating the required behavioural change and not just an intention of the public to ‘do better’, they are clearly supporters of the potential of zoos to create positive behaviour change and supply many persuasive arguments/examples.

After careful consideration of how good zoos demonstrate recreation, research, conservation and education, the authors advocate for a fifth role: animal welfare. This argument is established in both the zoo industry and in academia. The authors advocate for welfare to be *the* priority for modern zoos, for only if welfare is a priority and truly at the forefront of all zoo operations can a zoo be *for animals.* Previous authors have argued that welfare should refer to the collective well-being of all human and non-human animals connected with the zoo (Rose & Riley 2022), and there is exciting work by Justine Partoon *et al.* (2025) promoting the need for zoos to adopt an all-encompassing top-down welfare strategy which evidences good welfare practice at every organisational and operational level of the zoo. While these aspects are not discussed by explaining what good welfare is, and a threshold of acceptable welfare, the authors provide helpful context to the reader. Ultimately, the authors argue that zoo animals may have better welfare than their wild counterparts and, if true, justification for zoos no longer seems necessary, in part as this mitigates any welfare vs conservation argument. Animals who should not be kept in zoos on welfare grounds are discussed as are challenging topics like culling, with no easy solution found. By evidencing good welfare, all other roles of modern zoos are enhanced and, the authors argue, even greater justification for zoos exist.

*What Are Zoos For?* not only provides an account of what zoos are but also what they could be. At its core, this book provides a set of concepts and possibilities that all zoos should aspire to evidence. The book will give hope to existing advocates of the zoo industry, and maybe even reignite public passion for zoos after the daunting COVID years. For abolitionists, however, one question will remain, if this is what all zoos could be, why aren’t they? And this is a question that permeates with both author and reader. From the very first pages, Browning and Veit condemn poor zoos focused on profit and which do not prioritise animal welfare, with a bold and welcome statement that ‘*….they should be made illegal and shut down*’. There is consistent critique of zoo practice throughout this book. For example, in Chapter 3, the authors highlight that all zoos which are accredited need to ‘*shift focus*’ or ‘*make improvements*’ to be considered an ‘*empirical [research focused] zoo*’. What is missing, and I think deliberately so, is the grey area – the zoo that, on the surface, looks good, meets minimal welfare standards and evidences the policies and procedures of accrediting bodies, and is well-patronised, but where chronic welfare issues persist. While this book provides zoos with multiple ideas to achieve goodness and justify their own existence, it does not address the scope or scale of ‘okay’ zoos.

In summary, this is an accessible and well-argued justification for the existence of zoos; an interesting, honest and thoughtful take on what zoos could and should be.
